# Vaccinations and Immune Response in Celiac Disease

**DOI:** 10.3390/vaccines8020278

**Published:** 2020-06-05

**Authors:** Stefano Passanisi, Valeria Dipasquale, Claudio Romano

**Affiliations:** Paediatric Gastroenterology and Cystic Fibrosis Unit, Department of Human Pathology in Adult and Developmental Age “Gaetano Barresi”, University of Messina, 98124 Messina, Italy; spassanisi87@gmail.com (S.P.); dipasquale.valeria@libero.it (V.D.)

**Keywords:** adaptive immunity, children, hepatitis B, immunogenicity, pneumococcus, revaccination, rotavirus, vaccine

## Abstract

Immune response to vaccinations in celiac patients is of growing scientific interest. However, some aspects of the relationship between celiac disease (CD) and vaccines are still unclear. A comprehensive search of published literature using the PubMed database was carried out using the following key terms: “adaptive immunity”, “celiac disease”, “humoral immune response”, “immunization”, and “vaccination”. To date, there is no evidence showing any causative association between vaccines and CD development. Therefore, vaccinations may be administered according to the modalities and timing of the National Immunization Schedule for each country. The rotavirus vaccine is currently recommended for the general population, and according to some data, it appears to reduce the risk for the development of CD autoimmunity in the early years of life. Regarding the hepatitis B virus, a booster dose of the vaccine is often required due to the low or the lost immune response rate in CD. Furthermore, determination of hepatitis B antibody titers could be useful in newly diagnosed CD subjects regardless of age at diagnosis. Finally, pneumococcal vaccines may be administered in patients with advancing age at diagnosis and concomitant risk factors. Future clinical practice guidelines for vaccination and monitoring programs in celiac patients could be recommended.

## 1. Introduction

Celiac disease (CD) is one of the most frequent autoimmune diseases, occurring in about 1% of the Western population [1]. CD develops in genetically predisposed subjects who, in response to multiple environmental factors, manifest an immune reaction that is triggered by the ingestion of gluten [2]. Genetic predisposition is closely related to the presence of human leukocyte antigen (HLA)-DQ2 or DQ8 [3]. Recently, the European Society of Paediatric Gastroenterology, Hepatology and Nutrition updated and expanded evidence-based guidelines for diagnosing CD. The accuracy of the combination of total immunoglobulin (Ig) A and IgA antibodies against transglutaminase (TGA-IgA) and the safety of the no-biopsy approach for diagnosis in children with high TGA-IgA values (≥10 times the upper limit of normal) and positive endomysial antibodies in a second serum sample have been highlighted [4]. In recent decades, the prevalence of CD has been increasing, probably due to the greater availability of sensitive and specific screening tests [5]. However, it is also suggested that the steady rate of increase in diagnosis may be attributed to a true rise in incidence [6]. CD can occur at any age and has several clinical manifestations, varying from malabsorption to mildly symptomatic or non-symptomatic presentations. A gluten-free diet (GFD) remains the only recommended treatment. Although CD is a widespread and well-documented disease globally, some epidemiological, pathogenetic, clinical, and diagnostic factors are still unclear. Among these, one of the most intriguing aspects is the relationship between adaptive immunity and CD. Adaptive immunity, also known as acquired immunity, creates immunological memory after an initial response to a specific pathogen, and leads to an enhanced response to subsequent encounters with that pathogen. This process of acquired immunity is the basis of vaccination. Scientific interest in this topic arose in the 1970s, when early papers reported a large variety of immunological abnormalities in patients with CD [7,8,9]. Numerous clinical trials, meta-analyses and systematic reviews have since been conducted to better investigate the link between CD and vaccination [10,11,12]. However, a comprehensive vaccination program for CD patients is still missing.

In this manuscript, we provide an updated review of literature focusing on (i) the possible influence of vaccinations on CD onset, (ii) the immune response of celiac patients to vaccinations, and (iii) the recommended modalities and timing for any revaccinations. To accomplish this, we performed a comprehensive search of published literature using the PubMed MEDLINE database from inception to April 2020. We used the following combination of MeSH terms: “adaptive immunity (ID D056704)”, “celiac disease (ID D002446)”, “humoral immune response (ID D056724)”, “immunization (ID D007114)”, and “vaccination (ID D014611)”. All of the vaccines (hepatitis B virus, rotavirus, etc.) were later included as search keywords, in combination with “celiac disease”. They included English- and Italian-language studies which were available as full text. Particular emphasis was placed on evidence-based guidelines and all high-quality studies (randomized controlled studies, observational studies, reviews, and meta-analyses). 

## 2. Vaccinations and Celiac Disease Development

In the past, vaccinations have been indicated as risk factors for autoimmune diseases, such as CD and type 1 diabetes, because of their effect on immune system modulation [13,14]. However, the potential association between CD and vaccinations has been poorly investigated. In 1995, Thompson et al. [15] performed the first study aiming to retrospectively evaluate the influence of measles vaccination on the development of CD and inflammatory bowel disease, by comparing the prevalence of both diseases between a group of 2541 vaccinated individuals and a birth cohort of 11,407 unvaccinated subjects. No difference in CD prevalence was found between the two groups (*p* = 0.63). Later, Myléus et al. [16] conducted a retrospective study to investigate whether changes in the national Swedish vaccination program could be related to changes in the incidence rate of CD during the so-called “Swedish CD epidemic”, which lasted from 1984 to 1996. This early vaccination program included diphtheria/tetanus toxoid vaccine, acellular pertussis vaccine, inactivated polio vaccine, conjugated Hemophilus influenzae type b (Hib) vaccine, attenuated measles/mumps/rubella vaccine, and for at-risk groups, vaccination against tuberculosis with live attenuated variant of Mycobacterium bovis (i.e., bacillus Calmette–Guérin vaccine). Children who underwent early vaccinations showed no greater risk of developing CD. (15) The international observational DIABIMMUNE study, including children with CD from Estonia, Finland and Russian Karelia, stated that differences in vaccination programs between countries were not likely to cause differences in autoimmunity [17]. 

Wild-type rotavirus (RV) infection has been identified as a potential risk factor for the development of CD [17,18]. Notably, the study by Simre et al. [17] found that a gastrointestinal infection increased the risk of CD autoimmunity (presence of tissue transglutaminase autoantibodies) by 33% within the following 3 months. Therefore, RV vaccination could play a crucial role in reducing the risk for the development of CD autoimmunity in the early years of life. The potential protective role of oral attenuated RV vaccination has been studied by Vaarala et al. [19], who found a trend towards a lower incidence of CD in the cohort of vaccinated individuals during 4–6 years of follow-up after vaccination. Recently, a Finnish study found that the RV vaccine significantly reduced the risk of CD in childhood and adolescence. This study had a longer follow-up time (11–14 years after vaccination) than the Vaarala study, which probably allowed accumulation of a sufficient number of CD cases [20].

Kårhus et al. [21] examined the risk of CD following pandemic influenza vaccination in a cohort study of 2.6 million Norwegian people. The examined vaccine was the AS03-adjuvanted influenza A (H1N1) pdm09 vaccine. Although this study showed a slightly increased risk of CD diagnosis after pandemic influenza vaccination, no real causal effect of the vaccination has been demonstrated. 

A Scandinavian study including more than 3 million Danish and Swedish adult women showed a 56% increased risk of CD after human papillomavirus (HPV) vaccination. Notably, approximately half of all CD cases occurring after vaccination were diagnosed within one year of the first dose. Unmasking of underreported CD in HPV-vaccinated Scandinavian women could be a possible explanation for the increased relative risk [22].

To date, there is no evidence showing a causative association between vaccines and CD development. In addition, children with CD have no different contraindications to vaccinations than healthy peers. Therefore, vaccinations should be administered according to the modalities and timing of the National Immunization Schedule for each country. 

## 3. Vaccination Immunogenicity

CD patients have an increased risk of infections and secondary hospital admissions, which is related to several factors (e.g., defective nutritional status, increased intestinal permeability, hyposplenism) [11]. As is known, vaccinations play a crucial role in the prevention of infectious disease. However, vaccination immunogenicity in CD patients is uncertain. We therefore collected data on the immune response to various vaccines in CD patients. The sections concerning hepatitis B vaccine (HBV) are more thorough than those concerning other vaccines, due to greater availability of literature data.

### 3.1. Hepatitis B

An immunological response to HBV is estimated to occur in more than 90% of healthy vaccinated individuals [23,24]. Lack of response has been correlated with age, smoking, obesity, male gender, and the presence of specific HLA molecules, including HLA-DQ2, DR3, and DR7 [25]. A 2015 meta-analysis [10] concluded that CD patients had a significantly lower rate of protective antibody response to HBV vaccination than healthy controls. An evaluation of the response to HBV should be implemented as a routine assessment in newly diagnosed patients who had previously been vaccinated [11]. 

We analyzed both retrospective and prospective pediatric studies focusing on the immunological response, expressed by hepatitis B surface antibody (HBsAb) titers, available thus far. The response rate to HBV varied from 47% to 78%. In studies that compared a cohort of children with CD to a control group, a significant difference in the response to HBV vaccination was constantly found between the two groups [12,26,27,28,29,30,31,32,33,34,35,36,37] (Table 1). Wanting to clarify if age was a risk factor, Filippelli et al. [35], by comparing the percentage of responders and non-responders among three age groups (0–5.5 years, 5.5–9.5 years and 9.5–17 years), found no significant differences between the youngest and the oldest group. The data on adult patients also confirmed the high failure rate of HBV vaccination in CD subjects [38,39,40].

#### 3.1.1. Mechanisms Impairing the Immune Response

Different theories have been postulated to explain the poor response to HBV vaccination. The HLA system seems to contribute to the genetic susceptibility that impairs the immune response to vaccines [41]. Particularly, homozygosis for the HLA-DQ2 genotype may play a crucial role in the predisposition to weaker response to recombinant HBV in celiac patients [11]. In addition, HLA antigens may influence the clinical course of hepatitis B and hepatitis D superinfections [42]. Nevertheless, Filippelli et al. [35] in their prospective study reported no statistically significant difference regarding the comparison of the distribution of vaccine response between the different genotypes (DQ2/DQ2, DQ2/DQ8, and other HLA alleles).

Several studies have proposed gluten intake as a potential cause of failure of immunity [26,28,30,32]. Gluten intake could influence immune response due to the competition of both gliadin peptides and hepatitis B surface antigen (HBsAg) protein fragments for binding to HLADQ2 molecules, which could result in defective antibody production [26]. To assess the HBV vaccination response in relation to gluten exposure status, Zingone et al. [40] measured HBsAg and HBsAb titers among three groups of 163 CD patients (group A, 57 patients exposed to gluten; group B, 46 patients not exposed to gluten; group C, 60 infants) and 48 healthy controls (group D). An inadequate response to hepatitis B immunization was found in the three groups of CD patients compared to the control group (group A vs. controls, *p* < 0.001; group B vs. controls, *p* = 0.002; group C vs. controls, *p* = 0.001). Furthermore, no significant differences for group A versus group B and group A versus group C were evident. Moreover, in a recent retrospective study on CD pediatric patients who had previously been immunized for HBV as infants, the rates of low HBsAb concentration were not influenced by being in clinical remission on a GFD. The authors reported that the only factor that correlated with HBsAb concentration was age at time of HBsAb measurement, with an inverse association [37]. Indeed, time elapsed since HBV vaccination may play a crucial role in failure to respond to the vaccine. Lower immune responses were observed in CD patients aged 25–30 years, suggesting that the increase in the response failure rate in young adults could be related to the long interval between vaccination and HBsAb measurements [36]. In another retrospective study, the time between completion of vaccine series and diagnosis of CD was an independent predictor for lack of response (*p* = 0.021; OR = 0.69; CI 0.50–0.95) [34]. This finding supports the theory that a longer period between completion of vaccination and diagnosis of CD could increase the risk of vaccination failure [43].

Another factor that has been proposed as a reliable biomarker able to elucidate the causal relationship between immune response and CD is the high mobility group box 1 protein (HMGB1), which acts as an inflammation marker. It belongs to the alarmins family, promoting an immediate immune response to tissue damage [44]. Manti et al. [45] found an inverse association between serum HMGB1 levels and HBsAb concentrations in 49 pediatric patients with CD, suggesting that HMGB1 may represent a new molecule able to reflect clinical expression of the disease as well as immune impairment, resulting in HBV vaccination failure. 

#### 3.1.2. Management Strategies for Non-Responders

Non-responder celiac patients may represent a large reservoir of HBV-susceptible people leading to the spread of hepatitis B disease. Because of the predisposition to losing the immune response to HBV vaccination, a booster dose of vaccine has also been suggested in CD patients every 10 years regardless of their “unresponsiveness” status [46]. However, the modalities of revaccination (single booster or a complete schedule) and the route of administration (intramuscular (IM) or intradermal (ID)) are debated. The short-term immunogenicity of a pre-S vaccine was compared to a traditional HBV vaccine (Engerix B) as a possible strategy for revaccination of seronegative patients with CD who were previously immunized [47]. Pre-S vaccine is a recombinant hepatitis B vaccine, containing both the major S protein and the minor pre-S1 and pre-S2 proteins of the HBV coat. Instead, traditional hepatitis B vaccines consist only of the S protein component of the HBV surface antigen particle. Both vaccines elicited adequate booster responses after the first administration, suggesting that a single dose of both vaccines could be sufficient to obtain protective HBsAb titers in most CD patients [47]. A recent review aiming to investigate approaches in the event of non-response to HBV vaccination in CD patients showed that re-administering the vaccination series with the same dose of 10 or 20 μg (IM formulation) increased the rate of seroconversion to 67.5% in celiac subjects [48]. Rousseff reported a serological response after a single IM booster vaccination in 22/34 (65%) of previously non-responder celiac patients [33]. A systematic review and meta-analysis of randomized trials showed that IM vaccination was slightly more likely to achieve seroprotection than ID vaccination. Furthermore, this analysis highlighted that ID vaccination was mostly equivalent to IM vaccination in school-age children, suggesting that the ID route of vaccination administration might be more immunogenic in younger populations [49]. Indeed, Leonardi et al. [50] revaccinated 58 non-responder pediatric CD patients with ID (2 μg) or IM (10 μg) vaccine for a maximum of three booster doses, to compare the safety and efficacy of these two different vaccination routes. The authors found a similar percentage of “responders” after single (76.7% ID vs. 78.6% IM) and after 3 injections (ID = 90% vs. IM = 96.4%), although they documented a higher percentage of patients with an anti-HBs titer >1000 IU/L in the ID (40%) than in the IM (7.1%) group. 

### 3.2. Other Vaccines

Leonardi et al. [31] compared the immune response to vaccines against poliomyelitis, diphtheria, tetanus, measles, mumps, rubella, and pertussis between children with CD (*n* = 66) and age- and sex-matched control subjects (*n* = 50). The authors found no significant differences between the two groups. Similar findings were found by Park et al. [12] by comparing the response to rubella, tetanus and Hib vaccines between a cohort of 26 children with CD and a control group (*n* = 18). Another recent study found no significant differences in the percentage of responders to the first dose of measles vaccine between CD patients (*n* = 35) and the control group (*n* = 79). The median interval between the third dose of HBV vaccine and serum collection was 6.8 and 4.7 years for CD and control groups, respectively [33]. 

Regarding the hepatitis A virus (HAV) vaccination, Sari et al. [51] examined the immunogenicity of inactivated HAV vaccine in 33 children with CD compared to a sex- and age-matched control group (*n* = 66). Seroconversion rates were assessed 1 and 7 months after vaccination. The authors reported that pediatric celiac patients had a good immune response to the HAV vaccine, similar to healthy controls (21.2% vs. 22.6% of non-responders after 1 month, and 3% vs. 1.6% of non-responders after 7 months). By contrast, a Turkish study performed on 16 pediatric patients suffering from CD revealed lower immunological response to HAV vaccine in comparison to 50 healthy controls over a 7-year follow-up period (75% vs. 100%, respectively; *p* = 0.007) [32]. 

A prospective study on vaccination against influenza A/H1N1/09 among children with CD (*n* = 14) and age-matched healthy controls (*n* = 14) showed that CD patients achieved protective antibody titers comparable to the control group [52]. 

## 4. Pneumococcal Vaccination

The correlation between vaccination against streptococcus pneumoniae, also known as pneumococcus, and CD is of growing scientific interest. After the introduction of conjugate vaccines, the rate of pneumococcal disease decreased considerably among children in the vaccine target and among unvaccinated children, and adults [53]. To date, the commercially available pneumococcal vaccines are mainly 23-valent pneumococcal capsular polysaccharide vaccine (PPV 23) and 13-valent pneumococcal conjugate vaccine (PCV-13). PPV 23 produces opsonizing anti-capsular antibodies that confer protective action by a T-independent mechanism and is currently recommended in asplenic/hyposplenic adults and children >5 years [54]. PCV-13 contains a carrier protein, called CRM197 diphtheria protein, which changes the nature of the immune response from T-independent to T-dependent [55]. Furthermore, PCV-13 might also be useful in adult subjects with asplenia due to its T-dependent mechanism, which should not be compromised in this clinical condition. As recommended by the Advisory Committee on Immunization Practices, pneumococcal vaccination is mandatory in most countries for children <2 years, with PCV-13 [56]. In the following years, no further vaccine boosters are needed in the absence of risk factors (e.g., congenital immunodeficiencies, chronic renal failure, immunosuppressant diseases, sickle cell disease, and chronic heart disease) up to 65 years. 

Nevertheless, several reports of pneumococcal infection and fatal septicemia have been described in a number of celiac patients, particularly in the presence of spleen hypofunction [57,58,59,60]. Indeed, among all the various diseases associated with functional hyposplenism, CD is the most frequent [61]. 

To the best of our knowledge, there are no current data on the rate of CD patients who are vaccinated against pneumococcus. On the other hand, pneumococcal vaccination in adult CD patients appears to be drastically underused. Khan et al. [62] analyzed 119 celiac patients <65 years with at least one comorbidity, such as a concomitant autoimmune disease. The authors found that only 19.2% of these patients had been vaccinated against pneumococcus. Another study compared the risk of community-acquired pneumonia in both pediatric and adult celiac patients (*n* = 9803) and healthy controls (*n* = 101,755). The authors reported that only 26.6% of patients with CD had received pneumococcal vaccination after diagnosis. Furthermore, unvaccinated CD patients <65 had an increased risk of community-acquired pneumonia compared to unvaccinated individuals without CD (HR 1.28, 95% CI 1.02–1.60). This risk was higher at the time of diagnosis and for the following 5 years after diagnosis [63].

Authors have also proposed to investigate splenic function in CD patients at high risk of hyposplenism (e.g., patients with concomitant autoimmune disorders, old age at diagnosis, previous history of major infections/sepsis or thromboembolism, mesenteric lymph node cavitation, and/or spleen atrophy) [64]. Among available diagnostic tools, pitted red cell counting remains the most accurate, quantitative and inexpensive method, albeit observer-dependent [65]. 

## 5. Conclusions

To the best of our knowledge, there are no special recommendations on the approach to vaccinations in CD. Current evidence supports the absence of causal association between vaccinations against diphtheria/tetanus, pertussis, poliomyelitis, Hib, hepatitis B, measles, mumps and rubella, and CD development. These vaccines, which are mandatory in most countries around the world, may be administered according to the National Immunization Schedule for each country. In addition, RV vaccination may be strongly recommended by pediatricians since it can reduce the incidence of wild-type RV infections that have been identified as a potential risk factor for the development of CD autoimmunity in the early years of life. As far as vaccination against HBV is concerned, an approach might be to offer the determination of HBV antibody titers to newly diagnosed, previously vaccinated, CD subjects, regardless of age at diagnosis. However, since it is accepted that CD patients are predisposed to losing their antibody response to HBV vaccination, a booster dose of vaccine may be administered every 10 years regardless of the patients’ “unresponsiveness” status. A booster vaccination of HBV may be administered by the intradermal route in the younger population, and an intramuscular route may be used in adults. Pneumococcal vaccination may be administered in those patients with advancing age at diagnosis, concomitant autoimmune disorders, complications of CD, previous history of major infections/sepsis and/or thromboembolism, and splenic atrophy.

In conclusion, we recommend that CD patients should follow a regular vaccination schedule, regardless of age at diagnosis. As has been shown in many autoimmune diseases, a loss of response, especially for anti-hepatitis B vaccination should be monitored. Vaccinations against pneumococcus and rotavirus are recommended. 

## Figures and Tables

**Table 1:** Immune response to hepatitis B vaccine (HBV) vaccine in pediatric patients with celiac disease (CD).

Ref.	Year	Study Design	Population Characteristics	Response Rate *	Follow-up Period
[12]	2007	Retrospective	CD patients (*n* = 26, mean age 9.2 y) vs. controls (*n* = 18, mean age 10.4 y)	46.1% vs. 88.9% (*p* < 0.05)	6.4 vs. 6.9 y
[26]	2008	Retrospective	CD patients (*n* = 106, mean age 14 y) vs. controls (*n* = 113, mean age 14 y)	50.9% vs. 75.2% (*p* not available)	2.3 vs. 1.9 y
[27]	2009	Retrospective	CD patients (*n* = 60, mean age 9.3 y) vs. controls (*n* = 60, mean age 10.1 y)	50% vs. 88.4% (*p* < 0.0.001)	8 vs. 9 y
[28]	2010	Retrospective	CD patients (*n* = 40, mean age 12.4 y) vs. controls (*n* = 54, mean age 9.8 y)	47.5% vs. 85.2% (*p* < 0.05)	2.6 vs. 2.9 y
[29]	2011	Retrospective	CD patients (*n* = 64, mean age 4.7 y) vs. controls (*n* = 49, mean age 5.5 y)	78.1% vs. 95.9% (*p* = 0.001)	3.8 vs. 4.6 y
[30]	2011	Retrospective	CD patients (*n* = 52, mean age 10.7 y) vs. controls (*n* = 20, mean age 10.7 y)	61.5% vs. 90% (*p* < 0.05)	>6 vs. >6mo
[31]	2011	Retrospective	CD patients (*n* = 66, mean age 8.3 y) vs. controls (*n* = 50, mean age 7.6 y)	47% vs. 84% (*p* < 0.0001)	7 y vs. 6 y
[32]	2013	Prospective	CD patients (*n* = 30, mean age 6.2 y) vs. controls (*n* = 50, mean age 8.1 y)	70% vs. 90% (*p* = 0.03)	4 vs. 4 w
[34]	2014	Retrospective	CD patients (*n* = 53, mean age 9.6 y)	42%	9.3 y
[33]	2015	Retrospective	CD patients (*n* = 42, mean age 5 y) vs. controls (*n* = 79, mean age 7 y)	76.2% vs. 77.2% (*p* > 0.05)	3.5 vs. 4.7 y
[35]	2016	Prospective	CD patients (*n* = 49, mean age 6.8 y)	69.4%	Not specified
[36]	2019	Retrospective	CD patients (*n* = 133, mean age 7.3 y)	45%	Not specified
[37]	2020	Retrospective	CD patients (*n* = 373, mean age 6.7 y)	46.4%	Not specified

CD: celiac disease; mo: months; vs: versus; y: years. * Immune response evaluated by hepatitis B surface antibody (HBsAb) titers.
